# HIV-Related Behaviors, Social Support and Health-Related Quality of Life among Men Who Have Sex with Men and Women (MSMW): A Cross-Sectional Study in Chongqing, China

**DOI:** 10.1371/journal.pone.0118651

**Published:** 2015-02-20

**Authors:** Jiang-Peng Chen, Ming-Ming Han, Zi-Jun Liao, Zhen-Zhen Dai, Liang Liu, Hua Chen, Xiao-Yan Wen, Shan Hu, Ping Que, Wen Wen, Bin Peng

**Affiliations:** 1 Department of Health Statistics and Information Management, School of Public Health and Management, Chongqing Medical University, Chongqing, China; 2 Department of Epidemiology and Health Statistics, School of Public Health, Peking University, Beijing, China; 3 Beichuan Center for Disease Control and Prevention, Mianyang, Sichuan Province, China; Fudan University, CHINA

## Abstract

**Background:**

Health-related quality of life (HRQOL) has become commonly used both as a concept and as a field of research. However, little is known about the HRQOL of men who have sex with men and women (MSMW). The aim of this study was to examine HIV-related behaviors, social support, and HRQOL status and explore its predictors among MSMW.

**Methods:**

An anonymous cross-sectional study was conducted by snowball sampling method in 2013. A total of 563 Chinese MSM completed a structured questionnaire. The HRQOL and social support were measured with the Chinese version of the World Health Organization Quality of Life Scale (WHOQOL-BRFE) and the Social Support Rating Scale (SSRS), respectively.

**Results:**

Of the 563 MSM analyzed, 77 (13.68%) were MSMW who had a higher proportion of in-marriage and preference for an insertive role as compared with the men who have sex with men only (MSMO) (*P*<0.05). As high as 70.13% of MSMW had no regular sex partners and 72.73% of MSMW reported engaging in unprotected anal sex in the last six months. 36.36% had tested for HIV, while only 12.99% had accepted HIV voluntary counseling and testing (VCT) services. The scores of objective support and subjective support in MSMW were significantly higher than that of MSMO (*P*<0.05). No statistically significant difference was found in scores of all the four domains of the HRQOL between MSMW and MSMO. When comparing the HRQOL scores of MSMW with the Chinese general population reference group, the scores of MSMW were significantly lower in physical health domain. In a multivariate regression model, age, monthly income, sexual role, VCT acceptability, subjective support were associated with variability in HRQOL.

**Conclusions:**

To improve the HRQOL among MSMW, more attention needs to be paid to those with low social support, low-income, the old and those prefer a receptive role during anal sex populations.

## Introduction

As time passed, men who have sex with men (MSM) populations gradually manifested a high burden of HIV in many countries around the world. Despite varieties of interventions adopted, HIV prevalence was especially high in southwest China and sex between men has clearly become the main route of HIV transmission in mainland China [1]. The sentinel surveillance data showed that the HIV prevalence in Chongqing, a municipality in southwest China, has increased from 13.0% to 19.7% from 2006 to 2013, with an increase of 1% per year among MSM [2]. Low rates of condom use, multiple sexual partners and limited HIV testing were likely to increase the risk of HIV transmission. Although condoms are widely available and inexpensive, it is reported that rates of consistent condom use were still low [3] and condom use problems (i.e., breakage, slippage, or partial use (delayed application or early removal)) existed during anal sex [4].

Multiple sexual partnerships with both men and women are common among Chinese MSM and approximately 70–90% will eventually enter heterosexual marriage [5–7]. As a consequence, men who have sex with men and women (MSMW) represent an important target population for understanding the spread of HIV because of the inherent bridging aspect of their sexual behavior [8]. Most published studies have merely included MSMW in samples of “gay and bisexual men” or MSM, thereby obscuring the specific sexual health issues that MSMW face [9]. In America, several studies have noted that black MSMW have higher risk behaviors, including more sexual partners and lower rates of condom use, and higher HIV infection rates compared with black men who have sex with men only (MSMO) and with heterosexual men [10]. However, the place of MSMW framed as a potential “bridge” has not been paid enough attention to in HIV transmission in China. Preexisting risk factors and mediational mechanisms may operate differently among MSMW; therefore, it is extremely urgent to examine the disparities in HIV related-behaviors among MSMW and MSMO in China. In addition, the impact of HIV-related behaviors among MSMW on health-related quality of life (HRQOL) as a synthetical outcome remains unknown.

HRQOL is defined as “individual’s perception of their position in life in the context of the culture and value systems in which they live and in relation to their goals, expectations, standards, and concerns” [11]. The studies on HRQOL in MSM with HIV/AIDS have been widely conducted in context of developing countries [12–14]. However, the HRQOL among MSMW remains unknown. HRQOL is complicated by personal, environmental, and social factors. Therefore, the relationship between HIV-related behaviors and HRQOL was not clearly elucidated. Published studies have demonstrated that HIV-related behaviors were significantly associated with anxiety [15], depression [16] and the increased risk of HIV infection among MSM. Hence, it may also have an impact on their physical and psychological health which is a relatively important aspect of HRQOL.

Social support has captured the attention of both sociologist and medical scientists, which is a multi-construct with multiple dimensions. Cobb primarily defined social support as “information leading the subject to believe that he or she is cared for and loved, that he/she is esteemed and valued, and he/she belongs to a network of communication and mutual obligation” [17]. The relationship between social support and HRQOL among different types of special populations has been documented in the literature [18–21]. Nevertheless, few studies focused on the associations between social support and HRQOL among MSMW.

In conclusion, the present study was initiated to explore the HIV-related behaviors, social support, and HRQOL among MSMW, and to compare these with that of MSMO. The HRQOL in MSMW was also compared with the Chinese general population reference group. To develop targeted interventions, we assessed the influence of sexual role, MSM status disclosure, and other socio-demographic and HIV-related behavior characteristics and social support on HRQOL.

## Methods

### Study population

An anonymous cross-sectional study was conducted by snowball sampling method in 2013 in Chongqing city. Participants were eligible for the study if they were able to provide informed consent, 18 years of age or older, male at birth and reporting at least one male sexual partner in the last six months.

### Ethics statement

Ethical approval to undertake this study was granted by Chongqing Medical University ethics committee (2012010). Each participant received an information sheet, which documented the aim, procedures, potential threats, and benefits. Both written and verbal informed consents were obtained from the participants, immediately prior to the data collection.

## Measures

### Basic information

The demographic characteristics included age, occupation, monthly income, education level, MSM status disclosure, marital status, sexual role during anal sex. Existence of regular sex partners, frequency of condom use, HIV testing, HIV voluntary counseling and testing (VCT) services acceptability was also measured as HIV-related behaviors.

### Health-related quality of life (HRQOL)

HRQOL was assessed using the Chinese version of the World Health Organization Quality of Life Scale (WHOQOL-BREF) developed by WHO as a shortened version of WHOQOL-100. As described in a report from the WHOQOL Group, it is available in approximately 40 different languages for both developed and developing countries [22–23]. The Chinese version of the WHOQOL-BREF validated by Fang JQ et al [24] consisted of 28 items, including two items in the original WHOQOL-BREF assessing overall quality of life and general health satisfaction. The WHOQOL-BREF is a generic instrument with scores that are based on responses to individual questions, which are summarized into four domains: physical health (7 items), psychological health (6 items), social relationships (3 items), and environment (8 items). There were two items specific to Chinese: “Does family friction affect your life?” and “How is your appetite?” which were put at the end of the instrument. Each item is based upon self-report using a 5-point Likert scale. The response options range from 1(very dissatisfied/very poor) to 5 (very satisfied/very good). Raw domain scores for the WHOQOL were transformed to a 4–20 score according to guidelines [25]. Higher values indicate a better HRQOL, with the exception of three negative forms which include pain and discomfort, need for medical treatment, and negative feelings. The overall Cronbach’s alpha coefficient of the WHOQOL-BREF was 0.887. The coefficients for each of its domains were: physical health (0.557), psychological health (0.634), social relationships (0.63), and environmental (0.813).

### Social support

Social support was measured by the Social Support Rating Scale (SSRS) developed by Xiao [26]. SSRS is composed of 10 items grouped into three subscales: objective support (3 items), subjective support (4 items), and support utilization (3 items). Items were mostly scored on 4-point Likert scales (excluding questions asking for total number of “sources of support”), ranging from 1(never seek help from others) to 4(actively seek help from others). The scores for the scale range from 12 to 66, with higher scores reflecting more social support derived. The overall Cronbach’s alpha coefficient for SSRS in this study was 0.66. The coefficients for each of its domains were: objective support (0.424), subjective support (0.569), and support utilization (0.528).

### Statistical analysis

Statistical analyses were carried out using the SAS version9.3 (SAS Institute, Cary, NC) statistical software. Data are presented as mean ± standard deviation (SD) for continuous variables, and n (%) for categorical variables. Wilcoxon rank tests and Chi-square tests were performed to examine the differences in demographic data and health-related behaviors among MSMW and MSMO. The differences in social support and HRQOL between MSMW and MSMO were determined by Student’s *t*-test, while the differences between MSMW and the Chinese general population reference group were examined with *u*-tests. Multivariate stepwise linear regression analyses (with the four HRQOL domains and total HRQOL as the dependent variables) were performed to investigate the relationships between HRQOL scores, social support scores, HIV-related behaviors and socio-demographic variables. Relationships were considered statistically significant when *p*-values <0.05 in univariate analysis and *p*-values <0.10 in multivariate analysis were reached.

## Results

### Social-demographic characteristics

A total of 563 MSM participated in this study, 77 of whom were MSMW. An overview of the sample characteristics is presented in Table 1. The mean age of the MSMW was 26.42 years (SD = 6.36), with a range from 18 to 47 years old. It is important to note that monthly income was utilized as a proxy for potential socioeconomic status, with a larger portion of the sample (37.66%) reporting a monthly income between 1500 and 2999. A small proportion of respondents were students (9.09%), while the others were all employed. 61.04% had graduated from the college. 63 of 77 had never been married. Only 9 of 77 had disclosed their MSM status. Among the 77 eligible respondents, 10.39% were receptive role, while 37.66% respondents preferred an insertive role during sexual activity. There were no significant differences between MSMW and MSMO in socio-demographic characteristics except for the marital status and sexual role, which represented good comparable results in HIV-related behaviors, social support and HRQOL status.

**Table 1:** Social-demographic characteristics of study population.

**Characteristics**	**MSMW (n, %)**	**MSMO (n, %)**	***Z/χ*^2^**	***P***
**Age(years)** ^#^				
**<25**	38(49.35)	253(52.06)	1.08	0.2802
**25∼35**	30(38.96)	218(44.86)		
**≥35**	9(13.68)	15(3.09)		
**Occupation** ^*^				
**Student**	7(9.09)	81(16.67)	2.89	0.0890
**Employed**	70(90.91)	405(83.33)		
**Monthly income** ^#^				
**<800**	5(6.49)	33(6.79)	0.51	0.6077
**800∼1499**	9(11.69)	69(14.20)		
**1500∼2999**	29(37.66)	186(38.27)		
**3000∼4999**	22(28.57)	120(24.69)		
**≥5000**	12(15.58)	78(16.05)		
**Education level** ^#^				
**Junior school & Low**	12(15.58)	11(2.26)	1.42	0.1543
**Senior school**	11(14.29)	99(20.37)		
**College**	47(61.04)	340(69.96)		
**Master & Above**	7(9.09)	36(7.41)		
**MSM status disclosure** ^*^				
**Yes**	9(11.69)	64(13.17)	0.13	0.7194
**no**	68(88.31)	422(86.83)		
**Marital status** ^*^				
**Never married**	63(81.82)	463(95.27)	28.28	<0.0001
**Married**	11(14.29)	10(2.06)		
**Divorced/Widowed/Others**	3(3.90)	13(2.67)		
**Sexual role** ^*^				
**Insertive**	29(37.66)	149(30.66)	16.77	0.0002
**Versatile**	40(51.95)	176(36.21)		
**Receptive**	8(10.39)	161(33.13)		

Data are shown as the number (%)

^#^Wilcoxon rank tests were used

*Chi-square tests were used.

### HIV-related behaviors

Table 2 depicts the disparities in HIV-related behaviors between MSMW and MSMO. With regard to the sexual behaviors, as high as 70.13% of MSMW had no regular sex partners and 72.73% of MSMW reported engaging in unprotected anal sex in the last six months. Only 36.36% had tested for HIV, while 12.99% had accepted VCT services. There were significant differences in all the HIV-related behaviors between MSMW and MSMO (*P*<0.05).

**Table 2:** HIV-related behaviors of study population.

**HIV-related behaviors**	**MSMW (n, %)**	**MSMO (n, %)**	***Z/χ*^2^**	***P***
**Had regular sex partner in the last six months** ^*^				
**Yes**	23(29.87)	210(43.21)	4.88	0.0272
**No**	54(70.13)	276(56.79)		
**The frequency of condom use in the last six months** ^#^				
**All**	21(27.27)	226(46.50)	4.12	<0.0001
**Mostly**	26(33.77)	165(33.95)		
**Occasionally**	14(18.18)	62(12.76)		
**Never**	16(20.78)	33(6.79)		
**Had tested for HIV** ^*^				
**Yes**	28(36.36)	343(70.58)	34.62	<0.0001
**No**	49(63.64)	143(29.42)		
**Had accepted VCT service** ^*^				
**Yes**	10(12.99)	306(62.96)	67.42	<0.0001
**No**	67(87.01)	180(37.04)		

Data are shown as the number (%).

^#^Wilcoxon rank tests were used

*Chi-square tests were used.

### Social support

MSMW reported an average score of 32.01, 8.06, 16.70, 7.24 for total support, objective support, subjective support, support utilization, respectively (Table 3). The scores of objective support and subjective support in MSMW were significantly higher than that of MSMO (*P*<0.05).

**Table 3:** Social support and HRQOL scores of the two groups.

**Subscales of social support and HRQOL**	**MSMW**	**MSMO**	**t**	***P***
**Objective support**	8.06±3.14	7.08±2.46	3.14	0.0018
**Subjective support**	16.70±4.92	14.23±3.90	4.97	<0.0001
**Support utilization**	7.25±1.86	7.08±1.85	0.75	0.4534
**Total support**	32.01±8.03	28.39±6.00	4.68	0.4534
**Physical health**	14.43±2.04	14.05±2.07	1.51	0.1306
**Psychological health**	13.71±2.42	13.39±2.31	1.15	0.2498
**Social relationships**	13.68±2.53	13.52±2.78	0.48	0.6302
**Environment**	12.32±2.36	12.57±2.43	-0.83	0.4051
**Total HRQOL**	13.45±1.85	13.32±1.95	0.55	0.5797

Data are shown as the mean±SD.

### Health-related quality of life

MSMW reported an average score of 13.45, 14.43, 13.71, 13.68, and 12.32 for total quality of life, physical health, psychological health, social relationships, and environment, respectively (Table 3). No statistically significant difference was found in scores of all the four domains of the HRQOL between MSMW and MSMO.

The Chinese general population reference group comprised of 777 health adults. The means values of the physical health, psychological health, social relationship, and environment domains and total HRQOL among the Chinese general population reference group were 15.10±2.30, 13.89±1.89, 13.93±2.06, 12.14±2.08, and 13.38±2.91, respectively [24]. When comparing the HRQOL scores of MSMW with the Chinese general population reference group, the scores of MSMW were significantly lower in physical health domain (Table 4).

**Table 4:** HRQOL scores of MSMW and the Chinese general population reference group.

**Domains of HRQOL**	**MSMW(n = 77)**	**Reference Group(n = 777)**	**t**	***P***
**Physical health**	14.43±2.04	15.10±2.30	-2.46	0.0140
**Psychological health**	13.71±2.42	13.89±1.89	-0.78	0.4384
**Social relationships**	13.68±2.53	13.93±2.06	-0.99	0.3207
**Environment**	12.32±2.36	12.14±2.08	0.72	0.4747
**Total**	13.45±1.85	13.38±2.91	0.21	0.8361

Data are shown as the mean±SD; Reference group: Chinese general population reference group.

### Factors influencing health-related quality of life

Table 5 shows socio-demographic characteristics, HIV-related behaviors, and social support associated with HRQOL in multivariate linear regression analysis. There were effectively no statistically significant associations between participants’ socio-demographic characteristics (i.e. occupation, education level, MSM status disclosure, and marital status), frequency of condom use, previous HIV testing, and regular sex partners in the four HRQOL domains and the total HRQOL among MSMW. However, age, monthly income, sexual role during anal sex, VCT acceptability, subjective support were associated with variability in HRQOL. The more subjective support the respondents had, the higher scores they obtained in all domains except for the physical health domain. The older, the lower scores obtained in psychological health domain. The respondents who had more monthly income, the higher scores they had in psychological health domain and total HRQOL. The respondents preferred a receptive role during sexual activity had significantly lower scores in physical health domain. MSMW who had accepted VCT service had higher scores in physical health domain, but inversely in environment domain.

**Table 5:** Standardized regression coefficients for factors associated with HRQOL among MSMW.

**variables**	**Physical health**	**Psychological health**	**Social relationships**	**Environment**	**Total**
**Subjective support**	NS	0.312	0.312	0.334	0.305
**Age**	NS	-0.204	NS	NS	NS
**Monthly income**	NS	0.292	NS	NS	0.190
**Sexual role**	-0.327	NS	NS	NS	NS
**Had accepted VCT services**	0.276	NS	NS	-0.199	NS
**Adjusted R** ^**2**^	0.113	0.194	0.085	0.119	0.144

*P*<0.10; NS: not significant.

## Discussion

The present study examined the HIV-related behaviors, social support, and HRQOL status in MSMW, and compared these with that of MSMO. The HRQOL among MSMW was also compared with the Chinese general population reference group. To develop targeted interventions, we assessed the influence of sexual role, MSM status disclosure, and other socio-demographic and HIV-related behavior characteristics and social support on HRQOL. To our knowledge, this was the first examination of social support and HRQOL among MSMW. We determined primary findings. First, MSMW had a higher in-marriage rate and were more likely to prefer an insertive role during anal sex. Second, MSMW were more likely to engage in the sexual risk behaviors, while the rates of health care utilization were low. Third, MSMW had higher scores in objective support and subjective support, as compared with the MSMO. Fourth, there were no significant differences in scores of all the four domains of the HRQOL between MSMW and MSMO, while the score of physical health for MSMW was lower than that of the Chinese general population reference group. Fifth, age, monthly income, sexual role, VCT service acceptability, and subjective support were correlated with HRQOL among MSMW. No significant associations were observed between other demographic characteristics, HIV-related behaviors and HRQOL.

To our surprise, there were no significant differences in scores of all the four domains of the HRQOL between MSMW and MSMO, while only the score of physical health for MSMW was lower than that of the Chinese general population reference group. This suggests that the main task of HRQOL promotion among MSMW is to improve physical health status. Although, the homosexuality was already removed from the Chinese classification and diagnostic criteria for mental disorders since 2001, it behaved like a mental disease called autism spectrum disorder [27] whose key symptoms do not include the impairments in physical health similarly. Hence, according to the multivariate linear regression analysis, designed interventions targeted for those men who prefer a receptive role during anal sex among MSMW are needed. In addition, popularization of the VCT services was once again emphasized in this study.

Consistent with the previous studies [28–29], age and monthly income were the socio-demographic factors found to make small, unique contributions to the variance in HRQOL. In this study, age had negative relationship with psychological health domain. As age increases, the psychological health impairs. Financial status was also a strong predictor of HRQOL. MSMW with better income level have no financial stress in daily life in case of medical emergency, better living, and social activities which improve their HRQOL. Sexual role affects HRQOL in MSMW. Those who prefer a receptive role during anal sex among MSMW have poor HRQOL as compared with the versatile and insertive role in physical domain. The major reasons for this are discomfort during anal sex as a receptive role and the unhealthy body resulted from the fear of HIV infection. Therefore, to improve the HRQOL, low-income, the old populations, and those prefer a receptive role during anal sex among MSMW should be targeted.

The role of social support in sexual risk behaviors among MSM has been widely researched. Previous studies [30–31] showed lack of social support was associated with increased risk of sexual risk behaviors. But its impact on HRQOL has not been well confirmed. This study demonstrated that MSMW had higher scores in objective support and subjective support, as compared with the MSMO. The results of this study also implied that the ability to perceive subjective support seemed to play a more important role than objective support in improving HRQOL. One potential mechanism is through the buffering properties of social support. Social support can buffer the effects of stressful life events. Therefore, it can help MSMW adjust to life. Individuals who have higher levels of perceived social support can manage the situation effectively, even in the most difficult situation, while lower levels of perceived social support contribute to poor outcomes [32]. In this study, objective support and support utilization were not found to be significant for HRQOL. This situation may be explained by the fact that most of the MSMW did not disclose their MSMW status, thus they would not be differentially treated by relatives and friends. This suggests that MSMW need support to facilitate and maintain subjective social support. Designing and applying social relationship programs should be considered for MSMW to rebuild social support systems and then the HRQOL of MSMW could be improved.

MSMW were more likely to engage in the sexual risk behaviors, while the rates of health care utilization were low. As expected, the VCT acceptability was verified to be related to the physical health and environment domains. Those who accepted VCT services have mostly tested for HIV. The negative results of HIV testing may reduce the psychological pressure of HIV infection and then better participate in recreation or leisure. The counselling procedure, as a part of VCT services, may increase the participants’ HIV knowledge and reduce sexual risk behaviors. This may explain the mechanism of how VCT acceptability affects physical domain rated by MSMW. Rates of attending VCT clinics were lower among MSMW than MSMO, with 10/77 of MSMW and over two-thirds of MSMO (62.96%) reporting ever having attended VCT clinics. This acceptability rate among MSMW was consistent with previous studies conducted in Beijing and Urumqi, China [33], but is significantly lower than that in Hong Kong [34]. The Chinese government's ‘Plan for HIV/AIDS Prevention and Control among Men Who Have Sex with Men in China, 2007–2010’ set an ambitious target of achieving a 50% or higher HIV testing rate among MSM by 2010, recommending that MSM receive HIV testing at least once per year [33]. Despite the ongoing expansion of the national ‘Four Frees and One Care’ program and support of the Gates Foundations which provides free voluntary HIV screening and HIV testing, the HIV testing rate remains as low as 36.36% among MSMW which is lower among MSMO (70.58%), suggesting that efforts to expand the scopes of HIV testing among MSMW still need to be intensified. The rate of HIV testing (36.36%) is higher than that of attending VCT clinics (12.99%). This may be resulted from that most MSMW remain unaware of where and how to seek VCT services. Among MSMW who had ever tested for HIV, more than 2 in 3 were tested in places other than VCT clinics, despite the fact that Chinese government policy mandates free HIV testing throughout the VCT system.

Due to traditional cultural and family values, Chinese MSM often married to conceal their homosexuality from family and friends. It was estimated that 70–90% will eventually marry [6]. However, we found that 72.73% of MSMW reported engaging in unprotected anal sex in the last 6 months which is significantly higher than that of MSMO (46.50%) in present studies and MSM in previous studies conducted in China (47.1% in 2010) [35]. This strongly suggests that bisexual behaviors among MSM represent a very significant channel of HIV transmission to the female population. As MSMW are more sexually active and risk-taking in their sexual behaviors, intervention programs that target MSMW to improve HIV awareness and sero-status disclosure before and after entering marriage are essential.

However, several limitations in the present study should be noted. First, the number of subjects recruited is relatively small, which may reduce the statistical power and result in the failure to identify the probable predictors of HRQOL. Second, the sample populations were all in Chongqing, which is not representative of the MSMW in China. Third, all measures were subject to recall bias as they were self-reports of preference and recent sexual behavior, however, is a limitation with all cross-sectional studies. A follow-up survey using a more representative and larger sample size is dedicated to substantiating these findings.

## Conclusions

Significant differences of HIV-related behaviors were observed between MSMW and MSMO based the comparable socio-demographic characteristics, which should been taken into account when implementing behavioral interventions aimed at preventing HIV/AIDS transmission among MSM as well as from MSM to the general population in China. To improve the HRQOL, low-income, the old populations, and those prefer a receptive role during anal sex among MSMW should be targeted. Moreover, designing and applying social relationship programs should be considered for MSMW to rebuild social support systems and then the HRQOL of MSMW could be improved. Nevertheless, the present study has several limitations. A follow-up survey using a more representative and larger sample size is dedicated to substantiating these findings.

## Supporting Information

S1 Dataset Data file containing raw data and variables used for the present publication. The coding of each variable in the Excel file is equal to the options’ order of each variable exhibited in the tables.
(XLSX)

## References

[1] Zhang L, Chow EP, Jing J, Zhuang X, Li X, He M, et al HIV prevalence in China: integration of surveillance data and a systematic review. Lancet Infect Dis. 2013; 13: 955–963. 10.1016/S1473-3099(13)70245-7 24107261

[2] Zeng G, Feng L, Ouyang L, Lu R, Xu P, Wu G, et al The dynamic trends of HIV prevalence, risks, and prevention among men who have sex with men in Chongqing, China. Biomed Res Int. 2014; 2014:602719 10.1155/2014/602719 24783216 PMC3982456

[3] Jin M, Yang Z, Dong Z, Han J. Correlates of consistent condom use among men who have sex with men recruited through the Internet in Huzhou city: a cross-sectional survey. BMC Public Health. 2013; 13:1101 10.1186/1471-2458-13-1101 24289100 PMC3912932

[4] D'Anna LH, Margolis AD, Warner L, Korosteleva OA, O'Donnell L, Rietmeijer CA, et al Condom use problems during anal sex among men who have sex with men (MSM): findings from the Safe in the City study. AIDS Care. 2012; 24:1028–1038. 10.1080/09540121.2012.668285 22519680 PMC3389178

[5] Ruan Y, Li D, Li X, Qian HZ, Shi W, Zhang X, et al Relationship between syphilis and HIV infections among men who have sex with men in Beijing, China. Sex Transm Dis. 2007; 34:592–597. 17325622 10.1097/01.olq.0000253336.64324.ef

[6] Zhang B, Li X, Hu T, Liu D, Shi T. HIV/AIDS interventions targeting men who have sex with men (MSM): theory and practice. Chin J STD/AIDS Prev Cont. 2000; 6:155–157.

[7] Chow EP, Jing J, Feng Y, Min D, Zhang J, Wilson DP, et al Pattern of HIV testing and multiple sexual partnerships among men who have sex with men in China. BMC Infect Dis. 2013;13:549 10.1186/1471-2334-13-549 24238403 PMC3840637

[8] Williams CT, Mackesy-Amiti ME, McKirnan DJ, Ouellet LJ. Differences in sexual identity, risk practices, and sex partners between bisexual men and other men among alow-income drug-using sample. J Urban Health. 2009; 86 Suppl 1:93–106. 10.1007/s11524-009-9367-2 19479381 PMC2705486

[9] Dodge B, Jeffries WL 4th, Sandfort TG. Beyond the Down Low: sexual risk, protection, and disclosure among at-risk Black men who have sex with both men and women (MSMW). Arch Sex Behav. 2008; 37:683–696. 10.1007/s10508-008-9356-7 18512140 PMC2566750

[10] Tieu HV, Spikes P, Patterson J, Bonner S, Egan JE, Goodman K, et al Sociodemographic and risk behavior characteristics associated with unprotected sex with women among blackmen who have sex with men and women in New York City. AIDS Care. 2012; 24: 1111–1119. 10.1080/09540121.2012.672723 22533637 PMC3704079

[11] The WHOQOL Group. The World Health Organization Quality of Life assessment (WHOQOL): position paper from the World Health Organization. Soc Sci Med. 1995; 41: 1403–1409. 8560308 10.1016/0277-9536(95)00112-k

[12] Nglazi MD, West SJ, Dave JA, Levitt NS, Lambert EV. Quality of life in individuals living with HIV/AIDS attending a public sector antiretroviral service in Cape Town, South Africa. BMC Public Health. 2014; 14:676 10.1186/1471-2458-14-676 24990360 PMC4227123

[13] Soares GB, Garbin CA, Rovida TA, Garbin AJ. Oral health associated with quality of life of people living with HIV/AIDS in Brazil. Health Qual Life Outcomes. 2014; 12: 28 10.1186/1477-7525-12-28 24581005 PMC3942772

[14] Tran BX. Quality of life outcomes of antiretroviral treatment for HIV/AIDS patients in Vietnam . PLoS One. 2012; 7:e41062 10.1371/journal.pone.0041062 22911742 PMC3401204

[15] Mimiaga MJ, Biello KB, Sivasubramanian M, Mayer KH, Anand VR, Safren SA. Psychosocial risk factors for HIV sexual risk among Indian men who have sex with men. AIDS Care. 2013; 25:1109–1113. 10.1080/09540121.2012.749340 23339580 PMC3708996

[16] Wilson PA, Stadler G, Boone MR, Bolger N. Fluctuations in depression and well-being are associated with sexual risk episodes among HIV-positive men. Health Psychol. 2014; 33:681–685. 10.1037/a0035405 24467260 PMC4123959

[17] Cobb S. Social support as a moderator of life stress. Psychosom Med. 1976; 38:300–314. 981490 10.1097/00006842-197609000-00003

[18] Zheng Y, Ye DQ, Pan HF, Li WX, Li LH, Li J, et al Influence of social support on health-related quality of life in patients with systemic lupus erythematosus. Clin Rheumatol. 2009; 28:265–269. 10.1007/s10067-008-1033-7 19002545

[19] Zhao C, Wu Z, Xu J. The association between post-traumatic stress disorder symptoms and the quality of life among Wenchuan earthquake survivors: the role of social support as a moderator. Qual Life Res. 2013; 22:733–743. 10.1007/s11136-012-0197-4 22674337

[20] Deng M, Lan Y, Luo S. Quality of life estimate in stomach, colon, and rectal cancer patients in a hospital in China. Tumour Biol. 2013; 34:2809–2815. 10.1007/s13277-013-0839-3 23681801

[21] Xu J, Ou L. Resilience and quality of life among Wenchuan earthquake survivors: the mediating role of social support. Public Health. 2014; 128:430–437. 10.1016/j.puhe.2014.03.002 24792190

[22] Skevington SM. Advancing cross-cultural research on quality of life:observations drawn from the WHOQOL development. World Health Organisation Quality of Life Assessment. Qual Life Res. 2002; 11:135–144. 12018737 10.1023/a:1015013312456

[23] Nedjat S, Montazeri A, Holakouie K, Mohammad K, Majdzadeh R. Psychometric properties of the Iranian interview-administered version of the World Health Organization’s Quality of Life Questionnaire(WHOQOL-BREF): a population-based study. BMC Health Serv Res. 2008; 8:61–68. 10.1186/1472-6963-8-61 18366715 PMC2287168

[24] Fang JQ. Measurement and application of quality of life Beijing: Beijing Medical University Publishing Company; 2000.

[25] World Health Organization WHOQOL-BREF: Introduction, administration, scoring and generic version of the assessment—field trial version Geneva: WHO; 1996.

[26] Xiao SY. Theoretical foundation and application of Social Support Rating Scale. J Clin Psychiatry. 1994; 4:98–100.

[27] Lin LY. Quality of life of Taiwanese adults with autism spectrum disorder. PLoS One. 2014; 9:e109567 10.1371/journal.pone.0109567 25299379 PMC4192352

[28] Okaka EI, Davies M, Ahmed M, Naidoo S, Naicker S. Impact of socio-economic factors on quality of life in patients on continuous ambulatory peritoneal dialysis in an african setting. West Afr J Med. 2014; 33:125–129. 25236829

[29] Jung YE, Seo HJ, Song HR, Woo YS, Yim HW, Sung HM, et al Factors associated with subjective quality of life in Korean patients with depressive disorders: the CRESCEND study. Qual Life Res. 2012; 21:967–974. 10.1007/s11136-011-0006-5 21927913

[30] Ayala G, Bingham T, Kim J, Wheeler DP, Millett GA. Modeling the impact of social discrimination and financial hardship on the sexual risk of HIV among Latino and Black men who have sex with men. Am J Public Health. 2012; 102 Suppl 2:S242–249. 10.2105/AJPH.2011.300641 22401516 PMC3477911

[31] Hoff CC, Chakravarty D, Beougher SC, Neilands TB, Darbes LA. Relationship characteristics associated with sexual risk behavior among MSM in committed relationships. AIDS Patient Care STDS. 2012; 26:738–745. 10.1089/apc.2012.0198 23199191 PMC3513980

[32] Vyavaharkar M, Moneyham L, Tavakoli A, Philips KD, Murdaugh C, Jackson K, et al Social support, coping, and medication adherence among HIV-positive women with depression living in rural areas of the southeastern United States. Aids Patient Care and STDs. 2007; 21:667–680. 17919094 10.1089/apc.2006.0131

[33] Zou H, Wu Z, Yu J, Li M, Ablimit M, Li F, Poundstone K. Internet-facilitated, voluntary counseling and testing (VCT) clinic-based HIV testing among men who have sex with men in China. PLoS One. 2013; 8:e51919 10.1371/journal.pone.0051919 23418417 PMC3572127

[34] Lau JT, Gu J, Tsui HY, Wang Z. Prevalence and associated factors of intention to participate in HIV voluntary counseling and testing for the first time among men who have sex with men in Hong Kong, China. Prev Med. 2013; 57:813–818. 10.1016/j.ypmed.2013.09.005 24045009

[35] Wang K, Yan H, Liu Y, Leng Z, Wang B, Zhao J. Increasing prevalence of HIV and syphilis but decreasing rate of self-reported unprotected anal intercourse among men who had sex with men in Harbin, China: results of five consecutive surveys from 2006 to 2010. Int J Epidemiol. 2012; 41:423–432. 10.1093/ije/dyr182 22253304

